# Supplementary material for: No evidence from complementary data sources of a direct glutamatergic projection from the mouse anterior cingulate area to the hippocampal formation
Source: eLife. 2023 Aug 7;12:e77364. doi: 10.7554/eLife.77364 (PMC10425170; doi:10.7554/eLife.77364)
Supplement: Transparent reporting form [file elife-77364-transrepform1.docx]

***eLife’s* transparent reporting form**

We encourage authors to provide detailed information *within their submission* to facilitate the interpretation and replication of experiments. Authors can upload supporting documentation to indicate the use of appropriate reporting guidelines for health-related research (see [EQUATOR Network](http://www.equator-network.org/%20)), life science research (see the [BioSharing Information Resource](https://biosharing.org/)), or the [ARRIVE guidelines](http://www.plosbiology.org/article/info:doi/10.1371/journal.pbio.1000412) for reporting work involving animal research. Where applicable, authors should refer to any relevant reporting standards documents in this form.

If you have any questions, please consult our Journal Policies and/or contact us: [editorial@elifesciences.org](mailto:editorial@elifesciences.org).

**Sample-size estimation**

- You should state whether an appropriate sample size was computed when the study was being designed
- You should state the statistical method of sample size computation and any required assumptions
- If no explicit power analysis was used, you should describe how you decided what sample (replicate) size (number) to use

Please outline where this information can be found within the submission (e.g., sections or figure legends), or explain why this information doesn’t apply to your submission:

As this study was an attempt to replicate a finding, no sample size calculation was carried out prior to the experiments. If any of the viral injections conducted showed a projection from the anterior cingulate cortex to dorsal hippocampus – the null hypothesis (of no direct connection being present) would have been rejected.

We have, however, repeated each tracing experiment 30 (anterograde) and 10 (monosynaptic rabies tracing, with 6 injections in dCA1 and 4 in vCA1) times respectively, and 15 for classical tracer Fast Blue, and all of them confirmed our hypothesis.

**Replicates**

- You should report how often each experiment was performed
- You should include a definition of biological versus technical replication
- The data obtained should be provided and sufficient information should be provided to indicate the number of independent biological and/or technical replicates
- If you encountered any outliers, you should describe how these were handled
- Criteria for exclusion/inclusion of data should be clearly stated
- High-throughput sequence data should be uploaded before submission, with a private link for reviewers provided (these are available from both GEO and ArrayExpress)

Please outline where this information can be found within the submission (e.g., sections or figure legends), or explain why this information doesn’t apply to your submission:

In this study the biological replicates were mice and technical replicates were multiple sections (anatomical experiments).

For anterograde tracing experiments a total of 30 biological replicates was analyzed. For monosynaptic rabies tracing experiments, a minimum of 4 biological replicates was achieved for every condition; in each case at least 4 technical replicates were counted for each mouse, but where ever possible a higher number of technical replicates was used. In the case of retrograde AAV tracing we used 8 biological replicates. For classical tracer Fast Blue 7 biological replicates were analysed.

These data are included in the manuscript in methods (number of mice) and the corresponding figure legends. We will also provide the source data upon request (.tiff files).

**Statistical reporting**

- Statistical analysis methods should be described and justified
- Raw data should be presented in figures whenever informative to do so (typically when N per group is less than 10)
- For each experiment, you should identify the statistical tests used, exact values of N, definitions of center, methods of multiple test correction, and dispersion and precision measures (e.g., mean, median, SD, SEM, confidence intervals; and, for the major substantive results, a measure of effect size (e.g., Pearson's r, Cohen's d)
- Report exact p-values wherever possible alongside the summary statistics and 95% confidence intervals. These should be reported for all key questions and not only when the p-value is less than 0.05.

Please outline where this information can be found within the submission (e.g., sections or figure legends), or explain why this information doesn’t apply to your submission:

Figure 1-2 and Figure 4 - 5 – anatomical data and thus no statistical analysis was applicable.

Figure 3 - the Allen Brain atlas data mining data used Wilcoxon signed rank tests to compare signals from ipsilateral and contralateral structures in the brain.

(For large datasets, or papers with a very large number of statistical tests, you may upload a single table file with tests, Ns, etc., with reference to sections in the manuscript.)

**Group allocation**

- Indicate how samples were allocated into experimental groups (in the case of clinical studies, please specify allocation to treatment method); if randomization was used, please also state if restricted randomization was applied
- Indicate if masking was used during group allocation, data collection and/or data analysis

Please outline where this information can be found within the submission (e.g., sections or figure legends), or explain why this information doesn’t apply to your submission:

Group allocation for monosynaptic rabies tracing was based on genotype: Emx1-cre mice were used as controls to test selectivity of the pseudotyped rabies tracing, and Emx1-cre:flTVA mice were used for experimental conditions. For anatomical tracing experiments, no randomization was used for the allocation of animals into groups for injection location: each animal served as its own positive control as we could compare neuronal projection patterns with those already established in the scientific literature.

**Additional data files (“source data”)**

- We encourage you to upload relevant additional data files, such as numerical data that are represented as a graph in a figure, or as a summary table
- Where provided, these should be in the most useful format, and they can be uploaded as “Source data” files linked to a main figure or table
- Include model definition files including the full list of parameters used
- Include code used for data analysis (e.g., R, MatLab)
- Avoid stating that data files are “available upon request”

Please indicate the figures or tables for which source data files have been provided:

The source data to generate each graph is located as follows: fig 1: extended table 1; figure 2: contained within figure; fig 3: attached source data file; fig 4: qualitative data, no quantification used; fig 5: contained within figure and supplementary figure.
